# Chemical Analysis of a Swedish Fossil, in Which a New Earth Was Discovered

**Published:** 1804-07-01

**Authors:** 

**Affiliations:** Berlin


### Chemical Analysis of a Swedish Fossil, *in which a new* Earth *was discovered. By* Mr. Klaproth, *of Berlin.*

THE fossil which makes the object of the present examination is found in the mine of Bastnaos, near Riddarhytta, in the Swedish province of Westmannland. The first description of it is given by Mr. Cronstedt in the Transactions of the Swedish Academy of Sciences, 1751, where he considers it as a species of iron ore, which he names Tungstein; but according to the later researches of D'Elhyar, it essentially differs from the true Tungstone or Scheele ore, and contains,

| | |
|---|---|
| Lime - - - - - - - - | 54 |
| Iron - - - - - - - - | 24 |
| Siliceous earth - - - - | 22 |
| | 100 |

To the authority of this analysis it is probably owing, that Mr. Kirwan refers it to his Ferricalcit. The fossil itself has a crimson colour drawing a little to pink brown, and it is found either massive or scattered in small pieces; on the fresh fracture it is faintly shining, rather a little fatty, and splits very finely. The fragments are angular and sharp edged; it is not transparent and makes a greyish white streak; its powder is reddish grey; it is half hard, brittle, and in a high degree heavy. The specific weight is, according to Cronstedt, 4,988, but after my researches, 4,660. In analysing this fossil I proceeded in the following manner.

I. 1. A piece of the fossil being put into a platina crucible and ignited in a red heat, lost about two per cent. of its weight, changing the red colour into brown.

2. One hundred grains of the levigated fossil were exposed in a platina crucible to a still stronger igniting heat, whereby the powder lost five grains of its weight, and became dark brown.

II. One hundred grains were mixed with two hundred grains of carbonated kali, and ignited in a platina crucible. The mixture would not melt, but came out of the fire as a very brittle red brown mass, which was triturated and washed with boiling water. The lixivial liquor passed through the filtrum without colour, and it remained also clear, on being neutralized with nitric acid, which showed

 that that the fossil contained no Scheele oxyd. No trace of containing an acid could likewise be discovered by the solutions of silver, mercury, lead, iron, or barytes. The powder which had been thus sufficiently edulcorated, was digested with nitric acid, and after the siliceous earth had been separated, a solution of caustic kali was added, and the liquor kept boiling. When I had separated the alkaline liquor by the filtrum, I neutralized it with muriatic acid, and mixed with it carbonated kali, but no precipitation or turbidness ensued.

III. 1. Two hundred grains of the levigated fossil were digested in the boiling heat with two oucnes of muriatic acid, to which was afterwards added one ounce of nitric acid. After the whole quantity, except the siliceous earth, had been dissolved, the liquor was filtrated, and the remaining siliceous earth being washed and ignited, weighed sixty-eight grains.

2. The straw-yellow solution being neutralised by carbonated ammonia till it became no more turbid, was mixed with succinate of ammonia, and every time this was added a white caseous precipitation was occasioned, which on stirring the liquor disappeared again, and only a pale red precipitate of succinate of iron was deposited. The precipitation of the last substance being carefully finished, it was collected, washed, dried, and strongly ignited. The oxyd of iron thus obtained weighed nine grains.

3. The clear solution, after being entirely freed from iron particles, was precipitated by carbonated ammonia, whereby no disengagement of gas took place. The precipitate being collected, edulcorated, and dried, had the appearance of a loose milk-white powder which weighed 168 grains. After it had been deprived of the carbonic acid and water, it appeared with a cinnamon colour, and its weight was now 109 grains.

4. The liquors used for the edulcoration were evaporated, and the ammoniacal neutral salts that were obtained, being volatilised at a moderate heat, only a small trace of muriatic neutral salt was left behind.

From the subsequent examination it will appear, that the earth, which makes the principal constituent of that fossil, essentially differs from any of the known earths in several properties, on which account I consider it as a *new earth,* or an earth of its own, unless we are informed of the contrary by future experiments. The characteristical property of this earth to receive a light brown colour by ignition has induced me to give it the name of *Ochroït earth (Ochroïta)* which is derived from the Greek word οχρος (flavescens) and to the fossil itself the mineralogical name of *Ochroïtes.*—100 parts of this Ochroïtes have yielded,

| | | | | |
|---|---|---|---|---|
| Ochroït earth, | III. | 3, | - - | 54,50 |
| Siliceous earth, | | 1, | - - | 34, |
| Oxyd of iron, | | 2, | - - | 4, |
| Water, &c. | I. | 2, | - - | 5, |
| Loss | | | - - | 2, |
| | | | | 100 |

The properties of this new earth are the following.

1. The Ochroït earth, on being precipitated from acids by carbonated alkalis, absorbs part of the carbonic acid, and on being dried it imbibes water; 100 grains of this earth precipitated by carbonate of ammonia, and dried, lost 23 grains when saturated with nitric acid; 100 grains of the same earth lost by ignition 35 grains, consequently the proportion is, in 100 parts,

| | |
|---|---|
| Earth - - - - - - | 65 |
| Carbonic acid - - | 23 |
| Water - - - - - - | 12 |
| | 100 |

2. When freed from water and carbonic acid, the earth shews always a cinnamon colour, which is not produced either by iron or manganese particles.

3. When exposed to the heat of a porcelaine furnace in a charcoal crucible it is not in the least altered.

4. It glows before the tubus fusorius with a luminous appearance, but is not dissolved by borax, and merely mixed with it, by which it loses its particular colour.

5. The carbonated earth easily dissolves with effervescency in acids, and the taste of those solutions is astringent. When diluted the solutions appear without colour, but in a concentrated state they receive an amethystine colour. The ignited earth however is slowly soluble by acids in the cold, and the solutions, particularly that with nitric acid, appear with an orange colour, which disappears on diluting them with water.

6. The solution of this earth in sulphuric acid crystallises; the crystals which appear in the middle of the vessel seem to be octaëdrous, and are of an amethystine colour, and with difficulty soluble in water; whereas those which are formed on the sides of the vessel appear in radiant needles, and dissolve much easier in water.

7. On adding a saturated solution of sulphat of soda to a concentrated and neutralized solution of the earth in nitric or muriatic acid, both solutions are decomposed, and a white precipitate, with difficulty soluble in water, is formed, consisting of a combination of the earth with a small portion of sulphuric acid. By boiling this precipitate with double its weight of carbonat of soda, the earth will be separated in a carbonated state, and by means of this proceeding it may be obtained pure, and freed from any heterogeneous mixture.

8. Sulphurous acid easily dissolves this earth, and yields needle-like crystals of a pale amethystine colour.

9. The solution of this earth in nitric acid has little tendency to crystallization.

10. The muriated solution however yields prismatic crystals which are soluble in spirit of wine, to which, when burning, they impart no particular colour.

11. The solution in acetic acid gives no manifest crystals but rather coagulates to a thick mass.

12. It is precipitated from its solution in nitric and muriatic acids by carbonated alkalis with a milk-white colour, which however becomes greyish-yellow, when the earth is precipitated by caustic alkalis.

13. The prussiate of kali precipitates this earth from its neutral solution with a milk-white colour, and the precipitate makes a clear solution with nitric and muriatic acid, but when the earth contains a trace of iron the solution gets a bluish tint.

14. Tincture of galls produces no change, as likewise water impregnated with sulphurated hydrogen.

15. The succinats precipitate the earths in white flakes.

16. Phosphat of soda produces a white precipitate which disappears on adding nitric or muriatic acid, which is likewise the case with tartrit of kali.

17. A considerable precipitate is also effected by oxalic acid and oxalata. The oxalat of Ochroït earth, which is thus produced, differs from the former precipitates by being insoluble in nitric and muriatic acid, whence a nearer affinity of the oxalic acid to the Ochroït earth is evident.

18. Fixed alkalis have no action on the Ochroït earth, and do not dissolve it.

19. Ammonia acts under certain circumstances on this earth, but in a weak degree. A solution of 100 grains of carbonated earth, which however seemed not to be entirely free free from iron, was precipitated by carbonat of ammonia, and at the same time supersaturated with it. A few days after, during which time the liquor was frequently shaken, it was separated by the filtrum, perfectly saturated with sulphuric acid, and exposed in the warmth. It became turbid, depositing a grey precipitate, which, collected and dried, weighed 1¼ grains. It was dissolved in nitric acid, and mixed with prussiat of kali, whereby a precipitate of prussiat of kali was produced, after the separation of which the carbonat of kali precipitated a small portion of the earth in white flakes from the rest of the liquor. This proceeding may be employed to discover and to remove the least trace of iron.

According to these results the Ochroït earth comes near to the gadolin or ytter-earth, as it makes, like this earth, a transition of the simple earth to the metallic oxyds. The properties in which it agrees with ytter-earth consist in forming reddish crystals with sulphuric acid, and in being precipitated by prussiats; but it differs by having no sweet taste when combined with acids, and by being only weakly acted upon by carbonat of ammonia, and by appearing with a light brown colour, when ignited; farther, by not dissolving in borax or in phosphates before the tubus fusorius, whereas ytter-earth melts with both to a clear pearl.

